# Non-destructive measurement of total phenolic compounds in Arabidopsis under various stress conditions

**DOI:** 10.3389/fpls.2022.982247

**Published:** 2022-09-02

**Authors:** Praveen Kumar Jayapal, Rahul Joshi, Ramaraj Sathasivam, Bao Van Nguyen, Mohammad Akbar Faqeerzada, Sang Un Park, Domnic Sandanam, Byoung-Kwan Cho

**Affiliations:** ^1^Department of Biosystems Machinery Engineering, College of Agriculture and Life Science, Chungnam National University, Daejeon, South Korea; ^2^Disruptive and Sustainable Technologies for Agricultural Precision (DiSTAP), Singapore-MIT Alliance for Research and Technology (SMART), Singapore, Singapore; ^3^Department of Crop Science, College of Agriculture and Life Science, Chungnam National University, Daejeon, South Korea; ^4^Department of Smart Agriculture Systems, Chungnam National University, Daejeon, South Korea; ^5^Department of Computer Applications, National Institute of Technology, Tiruchirappalli, India

**Keywords:** plant stress, total phenolic compounds, hyperspectral, prediction, non-destructive

## Abstract

Quantifying the phenolic compounds in plants is essential for maintaining the beneficial effects of plants on human health. Existing measurement methods are destructive and/or time consuming. To overcome these issues, research was conducted to develop a non-destructive and rapid measurement of phenolic compounds using hyperspectral imaging (HSI) and machine learning. In this study, the Arabidopsis was used since it is a model plant. They were grown in controlled and various stress conditions (LED lights and drought). Images were captured using HSI in the range of 400–1,000 nm (VIS/NIR) and 900–2,500 nm (SWIR). Initially, the plant region was segmented, and the spectra were extracted from the segmented region. These spectra were synchronized with plants’ total phenolic content reference value, which was obtained from high-performance liquid chromatography (HPLC). The partial least square regression (PLSR) model was applied for total phenolic compound prediction. The best prediction values were achieved with SWIR spectra in comparison with VIS/NIR. Hence, SWIR spectra were further used. Spectral dimensionality reduction was performed based on discrete cosine transform (DCT) coefficients and the prediction was performed. The results were better than that of obtained with original spectra. The proposed model performance yielded *R*^2^-values of 0.97 and 0.96 for calibration and validation, respectively. The lowest standard errors of predictions (SEP) were 0.05 and 0.07 mg/g. The proposed model out-performed different state-of-the-art methods. These demonstrate the efficiency of the model in quantifying the total phenolic compounds that are present in plants and opens a way to develop a rapid measurement system.

## Introduction

As the global population continues to increase, there will be a huge demand for food in the near future. Hence, it is important to increase food production. There are abundant sources of phytochemicals, nutrients, etc., in the food that we ingest. Phytochemicals are the compounds that are produced by the plants. The three major groups of phytochemicals are polyphenols, terpenoids, and thiols. In plants, dietary phytochemicals such as flavonoids and phenolic compounds are present in the vegetables, leaves, fruits, etc. (Liu, 2013).

In recent decades, a lot of research has been conducted on the biochemical properties and the role of phenolic compounds (Tsao, 2010; Ignat et al., 2011). In particular, research into the effects of phenolic compounds on human beings is of great interest (Seca and Pinto, 2018; Şirin and Aslım, 2019). The phenolic compounds are key in defense responses such as antioxidant, anti-aging, and anti-proliferative activities. The long-term intake of phenolic compounds can help in fighting against cancer and chronic diseases such as diabetes, cardiovascular disease (CVD), and impaired cognitive functions (Del Rio et al., 2013).

Recent developments in plant metabolomics have made advancements in mapping and screening phenolic contents. Mass spectrometry has been used for the metabolite imaging of tissues (Boughton et al., 2016). Based on chemiluminescence and fluorescence, microfluidic biosensors are used for the optical analyses of particular compounds in plant tissues (Pires et al., 2014). Matrix-assisted laser desorption ionization plates are used for laser pulse analyses of the plant tissues (Sturtevant et al., 2016). The targeted sample selection is facilitated by laser micro-dissection technology, and the selected samples can be analyzed using conventional metabolomic tools in the laboratory (Gong et al., 2017). Despite advancements in modern molecular methods, there is great demand for non-destructive, fast, and accurate methods that can be used to measure chemical compounds.

To test many samples in a short period of time and in a non-destructive way, remote sensing methods are required. Hyperspectral imaging (HSI) is a suitable technique that can be used for detecting the plant stress and also measuring secondary metabolites in plants. Recently, many studies have focused on the HSI system. For instance, Mertens et al. (2021) used HSI to monitor the drought-induced changes and photosynthetic efficiency in maize plants with both index-based and PLSR models. The performance of close-range hyperspectral images for early drought detection and classification was studied and evaluated by using visible near-infrared HSI (Dao et al., 2021). Asaari et al. (2019) analyzed the hyperspectral images to detect the drought stress and recovery in maize plants. The HSI system had been used to detect the storage time for strawberries (Gong et al., 2017). Liang et al. (2018) determined and visualized the various levels of deoxynivalenol in bulk wheat kernels using HSI. HSI has been used to identify the chlorogenic acid content in Flos Lonicerae (Wang et al., 2019). The HSI-based prediction of the starch content based on a single kernel in corn seeds has been performed (Liu et al., 2020). The protein content in a single wheat kernel has been predicted using HSI (Caporaso et al., 2018a). The moisture content and anthocyanin have been detected in purple potato slices using visible HSI (Tian et al., 2021). Erkinbaev et al. (2019) developed an artificial neural network model that was based on HSI to identify the hardness of wheat. The random forest model has been applied to study the bruising degrees of apples (Tan et al., 2018). Aflatoxin has been detected from peanuts using deep learning and HSI (Han and Gao, 2019). The machine learning method has been applied on black rice HSI data to predict the anthocyanin content (Amanah et al., 2021).

Although there have been multiple studies that have predicted the phenolic compounds that are present in peated barley malt (Yan et al., 2021), persimmon leaf (Mayranti et al., 2019), grape seeds and skins (Zhang et al., 2017), basil seeds (Choi et al., 2020), and chicory leaves (Sytar et al., 2020), applying HSI for the non-destructive measurement of the phenolic compound contents in plants that have been grown under various stress environments has not been fully explored. In addition to these research works, the presence of various amounts of phenolic compounds in plants under different stress conditions provides an opportunity to assess non-destructive HSI methods and their ability to identify the total phenolic compounds in stressed and normal plants. In our research study, the Arabidopsis thaliana plant was used since it is a model plant. The main focus of this study was to analyze the hyperspectral data in order to identify the total phenolic compounds that are present in plants. This study also shows that the use of HSI analysis to assess the induced phenolic content changes in aboveground plant parts and for rapid prediction paves the way toward the systematic production of bio-active compounds for nutraceutical use.

## Materials and methods

### Plant sample

**FIGURE 1:**
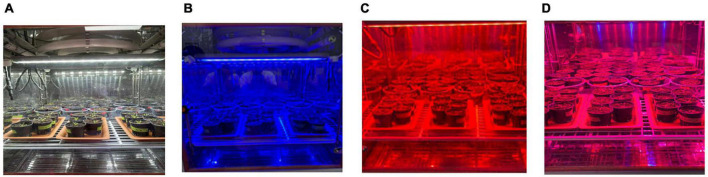
Plant growth in various light conditions. **(A)** white LED, **(B)** blue LED **(C)**, red LED **(D)**, and red–blue LED.

In our study, we chose Arabidopsis thaliana since it is a model plant that can be used for genetic studies, and it has a short life span. The Arabidopsis seeds had been soaked in medium. Once the seeds had been germinated, they were moved to chambers with four different light conditions (Figure 1)—white light (wavelength: 380 nm), red light (wavelength 640 nm), blue light (wavelength 430 nm), and red–blue light (Red:Blue ratio of 7:3)—for an 8 h photoperiod. The Arabidopsis plants were grown at 25 °C and in 70% humidity throughout the experiment. After a 2-week growth period in the abovementioned conditions, half of the plants had been placed under a drought stress (5 ml water per plant twice in a week) for a period of 1 week.

### NIR-HSI system

In our study, a line scan NIR-HSI system was used to collect the Visible/NIR (VIS/NIR) and Shortwave Infrared (SWIR) data of the plants. The VIS/NIR HSI system consisted of an EMCCD camera (Luca RDL-604M, Andor Technology, South Windsor, CT, United States), light sources, and a line scan imaging spectrograph (Headwall Photonics, Fitchburg, MA, United States). The spectral range of the VIS/NIR HSI system was 400–1,000 nm. The SWIR HSI system consisted of a line-scan spectrograph (NIR, Headwall Photonics, Fitchburg, MA, United States), six 100 W tungsten halogen light sources, a mercury cadmium telluride (MCT) detector (Model: Xeva-2.5–320; Xenics, Heverlee, Belgium) to detect the light reflection produced by the plant sample, a camera with a 320 × 256 pixel resolution, and a translation stage. The spectral range of the SWIR HSI system was 900–2,500 nm. Both the VIS/NIR and SWIR HSI systems were controlled by a computer with Windows OS.

### NIR-HSI plant data acquisition and extraction

The hyperspectral images/data (*I*_*h*_) of the plants were collected using an NIR-HSI system, which can be described as follows: A pot containing an Arabidopsis plant was kept over a moving table. The table moved from right to left and was controlled by a stepping motor. To cover the spatial range of the plants, the distance between the camera and the sample was set to 80 cm. The plant sample was scanned line by line with the help of the HSI system. When the plant samples passed the camera field of view (FOV), 3D hypercubes of the plant samples were obtained. In total, there are 120 scanned plant samples. To correct the environmental noise and to calculate the reflectance value, white (*I*_*w*_) and dark (*I*_*d*_) reference images were collected. The dark reference image (0% reflectance) was collected with the when the lights were switched off and when the camera lens was covered with an opaque cap. Additionally, the white reference image (>99% reflectivity) was collected using a white Teflon plate. The HSI-corrected image (*I*_*c*_) was obtained using following equation:


(1) $$I_{\text{c}}=\frac{I_{h}-I_{d}}{I_{w}-I_{d}}$$

**FIGURE 2:**
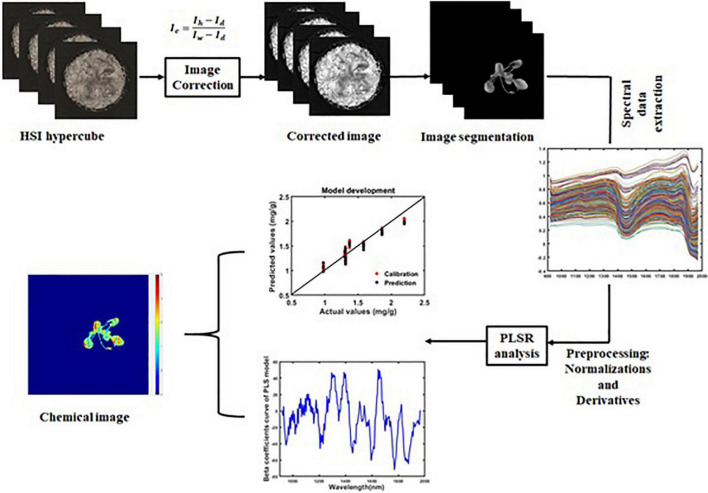
Workflow of phenolic content prediction in plants.

The HSI-corrected images (Ic) of plants contain background regions such as the pot, soil, etc. To extract the spectral information of each plant from those background regions, the plant region was segmented. While segmenting the plant region in the SWIR images, spatial data that corresponded to the wavelengths of 947, 1,535, and 1,946 nm were used. Similarly, for plant region segmentation from the VIS/NIR images, spatial data with the wavelengths 560, 655, and 750 nm were used. The spectral data were extracted from the segmented images. These processes were performed using MATLAB software (R2020b). The workflow of the phenolic content prediction process is given in Figure 2.

### Data pre-processing

The extracted spectral data may contain noise that was produced by the camera and by the environment. Preprocessing methods such as the normalization, multiplicative scatter correction (MSC), standard normal variate (SNV), and Savitzky–Golay (1st and 2nd derivative) methods were over the extracted spectral data. To compensate for the inconsistencies that were caused because of the optical source length and the sample thickness, the spectral data were fit within the range of (0–1) using the normalization method. The MSC preprocessing method was used to correct the scattering intensity of the spectra (Candolfi et al., 1999). To correct the baseline effect and to remove the overlapping peaks in the spectra, Savitzky–Golay (SG) derivatives were applied (Osborne et al., 1993).

### High-performance liquid chromatography for reference data

Since the Arabidopsis plants were all small in size and did not weigh much, all of the plants that were grown in the same growing conditions were analyzed in a group instead of individually. In our study, we grouped 15 plants into one growing condition (120 plants/8 conditions = 15 plants per condition). The protocol that was used to extract and analyze the phenylpropanoid compounds was explained in Park et al. (2019). A quantity of 100 mg of each fine powder sample was taken, and it was mixed with 80% aqueous MeOH solution. An amount of 3 ml of the MeOH solution was used for mixing. The mixture was vortexed for 1 min, and it was sonicated at the temperature of 37 °C for a period of 1 h. Then, the mixture was centrifuged at the speed of 10,000 rpm at the temperature of 4 °C for a period of 0.25 h. Then, the supernatants were gathered, and a 0.45 μm PTFE syringe filter (Millipore, Bedford, MA, United States) was used to filter sterilize them into an amber glass screw thread vials (Thermo Fisher Scientific, United States). The phenylpropanoid compounds were separated through a C18 column (5 μm, 0 × 4.6 mm) at the temperature of 30°C using an Agilent Technologies 1,200 series HPLC system (Palo Alto, CA, United States) at 280 nm. The mobile phase contained MeOH/water/acetic acid (5:92.5:2.5,v/v/v) (solvent A) and MeOH/water/acetic acid (95:2.5:2.5, v/v/v) (solvent B); an injection volume of 20 μL and a flow rate of 1.0 mL/min were used. The gradient program was as follows: 0% solvent B; 0–80% solvent B, 48 min; 0% solvent B, 10 min. Each phenolic compound was determined based on the retention time and spiking tests results. The quantification of the phenolic contents of each sample was performed with reference to a corresponding calibration curve.

### Model development

The partial least squares regression (PLSR) method was applied to predict the total phenolic contents in plants that had been grown in various conditions, as discussed earlier in this section. PLSR is a multivariate model that integrates multiple regression and feature-based extraction over the principal component method and that can be used to identify the response of dependent variables based on a huge number of independent variables. The PLSR model is based on the linear relationship between the variables X and Y. This builds a possibility for predicting the X variable component (Kresta et al., 1994). The PLS model is represented by the following equations:


(2) $$X=A\,B^{T}+C$$

(3) $$Y=D\,E^{T}+F$$

(4) $$D=AG+H\;\left(G={\left(A^{T}A\right)}^{-1}A^{T}D\right)$$

where X is the spectral data and is an independent variable matrix, and Y denotes the total phenolic content in plants and is a dependent variable. A and D represent the score matrices, and B and E are the loading matrices of X and Y, respectively. C and F represent the error matrices for X and Y.

The relationship between the total phenolic content and the spectral data was developed using the least squares method, as given in Equation 4. This technique was recommended for this study since it was successfully used to predict phytochemical compounds in previous studies (Amanah et al., 2021).

In this study, for each of the segmented plant hyperspectral images, the plant regions were divided randomly into ten different regions, and the mean spectra for each region were calculated. The mean process was carried out to accommodate the physical characteristic differences within a sample (Amanah et al., 2021). The mean process resulted in 1,200 spectral data (10 spectra × 120 plant samples). This entire dataset was divided into calibration and validation datasets in the ratio of 70:30. The wavelength that was used for model development ranged from 400 to 990 nm for VIS/NIR and from 920 to 1,970 nm for SWIR. Other wavelengths were not considered in this study since they contain noisy regions curve.

In this study, the phenolic compounds have been predicted by selecting a few wavebands using Discrete Cosine Transform (DCT) coefficients. The DCT coefficients for each waveband have been calculated. Since the flat (DC) coefficient have more energy when compared with other coefficients, it is capable of reconstructing the original spectra (PraveenKumar and Domnic, 2020). Hence, it is consider as similar to original spectra and is not considered in our experiment. The remaining coefficients are known as AC coefficients. The energy of the coefficients is high in a few leftmost coefficients, decreases further and becomes less in a few rightmost coefficients. The high frequency AC coefficients (rightmost coefficients) are not considered since they represent the edge features (PraveenKumar and Domnic, 2020). Based on these properties, we considered the highest energy AC coefficient for selecting the wavebands in our experiment. Then, the peaks among the highest AC coefficients of all the wavebands have been identified and the corresponding wavebands are selected as the reduced wavebands. In our experiment, these wavebands have been used for phenolic prediction.

### Image processing

HSI can be used to create chemical images while predicting the chemical compounds that are in the sample. This helps us to view the chemical distribution throughout the sample. The beta coefficient of the developed model was used to create the chemical images. Initially, the 3D hyperspectral image was converted to a 2D matrix, and then this matrix was multiplied with PLSR beta coefficients. Finally, the resulting matrix was converted back to a 3D image. The visualization of the total phenolic compound concentrations in the plant region was conducted by summing the corresponding pixels of all of the band images. This can be represented by following equation:


(5) $$I_{c\,h}=\sum_{i=1}^{n}I_{i}\,K_{i}\,L$$

where I_ch_ is the chemical image, I_i_ represents the ith band of the hypercube, K_*i*_ denotes the PLSR beta coefficients, L is the constant, and n is the total number of bands.

## Results

### High-performance liquid chromatography result

The reference values for the total phenolic compounds in this study were evaluated from 120 plants grown in various light conditions, non-drought, and drought conditions, and the results are presented in Table 1.

**TABLE 1:** The reference values of phenolic compounds [mg/g dry weight (DW)] obtained from HPLC analysis.

S. No	Phenolic compounds	Growing conditions							
		White + Non-drought	White + Drought	Blue + Non-drought	Blue + Drought	Red + Non-drought	Red + Drought	RedBlue + Non-drought	RedBlue + Drought
1	Gallic acid	0.043 ± 0.010 a^1^	0.017 ± 0.002 b	ND	ND	ND	ND	ND	ND
2	Catechin	0.150 ± 0.011 a	0.145 ± 0.026 a	0.188 ± 0.033 a	0.137 ± 0.013 a	0.141 ± 0.003 a	0.165 ± 0.021 a	0.130 ± 0.014 a	0.134 ± 0.014 a
3	Chlorogenic acid	0.123 ± 0.005 a	0.137 ± 0.009 a	0.116 ± 0.019 a	0.122 ± 0.008 a	0.123 ± 0.002 a	0.119 ± 0.011 a	ND	ND
4	Caffeic acid	0.059 ± 0.005 b	0.050 ± 0.011 b	0.093 ± 0.011 a	ND	0.049 ± 0.010 b	0.060 ± 0.007 b	ND	ND
5	(-)-Epicatechin	0.055 ± 0.011 b	0.037 ± 0.004 b	0.082 ± 0.021 a	0.042 ± 0.006 b	ND	ND	ND	ND
6	Epicatechin gallate	0.743 ± 0.023 a	0.302 ± 0.030 b	ND	0.151 ± 0.018 c	0.124 ± 0.006 c	0.255 ± 0.030 b	ND	ND
7	Ferulic acid	0.138 ± 0.014 b	0.384 ± 0.029 a	ND	ND	0.033 ± 0.013 cd	0.053 ± 0.001 c	ND	ND
8	Sinapic acid	0.032 ± 0.002 a	0.035 ± 0.007 a	0.005 ± 0.001 c	ND	ND	0.015 ± 0.002 b	ND	ND
9	Benzoic acid	ND	ND	0.164 ± 0.013 a	0.137 ± 0.013 b	0.136 ± 0.002 b	0.138 ± 0.009 b	ND	0.135 ± 0.010 b
10	Rutin	0.39 ± 0.040 ab	0.390 ± 0.043 ab	0.324 ± 0.010 b	0.330 ± 0.006 b	0.340 ± 0.005 b	0.339 ± 0.018 b	0.464 ± 0.132 ab	0.600 ± 0.168 a
11	Quercetin	0.287 ± 0.018 a	0.259 ± 0.014 a	0.268 ± 0.018 a	0.271 ± 0.011 a	0.281 ± 0.004 a	0.339 ± 0.049 a	0.283 ± 0.016 a	0.354 ± 0.085 a
12	Kaempferol	0.174 ± 0.038 a	0.104 ± 0.016 b	0.131 ± 0.020 ab	0.110 ± 0.015 b	0.085 ± 0.008 b	0.098 ± 0.017 b	0.100 ± 0.014 b	0.086 ± 0.013 b
TOTAL		2.194 ± 0.053 a	1.859 ± 0.084 b	1.371 ± 0.017 c	1.300 ± 0.037 cd	1.311 ± 0.013 cd	1.582 ± 0.063 bc	0.977 ± 0.136 d	1.309 ± 0.241 cd

*ND denotes compound not detected.

^1^The different letters followed by the values in a column denote the significant difference (p < 0.005) between the parameter areas using Duncan’s multiple range test (n ≥ 3, mean ± SD).

The concentration of each phenolic compound given in Table 1 varies for each growing condition. Although the concentrations of certain phenolic compounds may be similar to one another in each growing condition, the individual phenolic content may differ significantly. As a result, total phenolic compound prediction is critical for plants that have been grown under various stress conditions. In addition, all of the phenolic compounds are important for plants as well as for human health, and this study focuses on predicting the total phenolic compounds.

### Model prediction result

In this study, a multilinear regression model was developed using 1,200 spectra ranging from 400 to 990 nm in the VIS/NIR region and by using 1,200 spectra ranging from 920 to 1,970 nm in the SWIR region. The total phenolic compound prediction results of the PLSR model (without band dimensionality reduction) for the that had been plants grown in various stress conditions are given in Table 2. The PLSR model shows acceptable performance for the spectra that were obtained from the VIS/NIR regions, as they obtained high correlation coefficients (*R*^2^) of 0.87 and 0.83 for calibration and validation datasets, respectively. The model performance for the spectra that were obtained from the SWIR regions are better than those that were obtained in the VIS/NIR region spectra, attaining the *R*^2^-values of 0.94 and 0.93 for calibration and validation datasets, respectively. Hence, the SWIR spectra is considered for further analysis. The model performance for the SWIR spectra of the validation dataset is closer to 0.9 (*R*^2^-value) for all of the preprocessing methods except MSC and SNV. The results of the total phenolic compound prediction with reduced number of wavebands using PLSR model is given in Table 3. It is observed from Table 3 that the prediction model attains the *R*^2^-values of 0.97 and 0.96 for calibration and validation datasets, respectively. These values are better than the prediction results obtained with full wavebands.

**TABLE 2:** PLSR model performance of total phenolic compound prediction in plants grown under various stress conditions (full waveband).

Pre-processing	VIS/NIR				SWIR			
	R_c_^2^	SEC	R_v_^2^	SEP	R_c_^2^	SEC	R_v_^2^	SEP
Mean normalization	0.84	0.17	0.44	0.27	0.93	0.11	0.89	0.14
Maximum normalization	0.84	0.17	0.51	0.25	0.93	0.11	0.89	0.13
Range normalization	0.83	0.17	0.5	0.26	0.88	0.14	0.85	0.16
MSC	0.79	0.19	0.62	0.27	0.63	0.25	0.24	0.36
SNV	0.80	0.18	0.82	0.17	0.85	0.16	0.71	0.22
SG_1st derivative	0.84	0.16	0.68	0.23	0.91	0.12	0.83	0.17
SG_2nd derivative	0.87	0.15	0.83	0.17	0.94	0.10	0.93	0.11
Raw	0.83	0.17	0.53	0.28	0.93	0.11	0.87	0.15

MSC, multiplication scatter correction; SNV, standard normal variate; SG, Savitzky–Golay; SEC, standard error of calibration; SEP, standard error of prediction in validation.

**TABLE 3:** PLSR model performance of total phenolic compound prediction in plants grown under various stress conditions (after waveband reduction).

Pre-processing	R_c_^2^	SEC	R_v_^2^	SEP
Mean normalization	0.89	0.11	0.81	0.16
Maximum normalization	0.84	0.14	0.75	0.19
Range normalization	0.80	0.16	0.73	0.19
MSC	0.75	0.18	0.57	0.24
SNV	0.82	0.15	0.65	0.22
SG_1st derivative	0.97	0.05	0.93	0.10
SG_2nd derivative	0.97	0.05	0.96	0.07
Raw	0.94	0.10	0.91	0.11

## Discussion

### The spectral characteristics

The plant spectral data in the wavelength between 400 and 990 nm for the VIS/NIR spectra and between 920 and 1,970 nm for the SWIR are characterized by several peaks. In VIS/NIR the spectra, the wavelength between 442 and 665 nm indicates the water status (Jopia et al., 2020). Similarly, the wavelength at around 780 nm is the region that indicates the water absorption (Zhang et al., 2012). The optimal wavelengths that contain phenolic compound information are from 450–475 nm to 535–565 nm (Mayranti et al., 2019). In SWIR, the water absorption region is in wavelength at 1,450 nm (OH stretch first overtone) (Aenugu et al., 2011). The presence of phenolic content can be identified in the wavelengths between 1,084 and 1,318 nm (CH second overtone) and between 1,609 and 1,861 nm (CH first overtone) (Ma et al., 2019). In Kokaly and Skidmore (2015), the phenolic content is identified by bands near 880, 1,130, and 1,660 nm. The presence of phenolic compounds is confirmed at the wavelengths between 1,110 and 1,130 nm and at around 1,650 nm (Yan et al., 2021).

**FIGURE 3:**
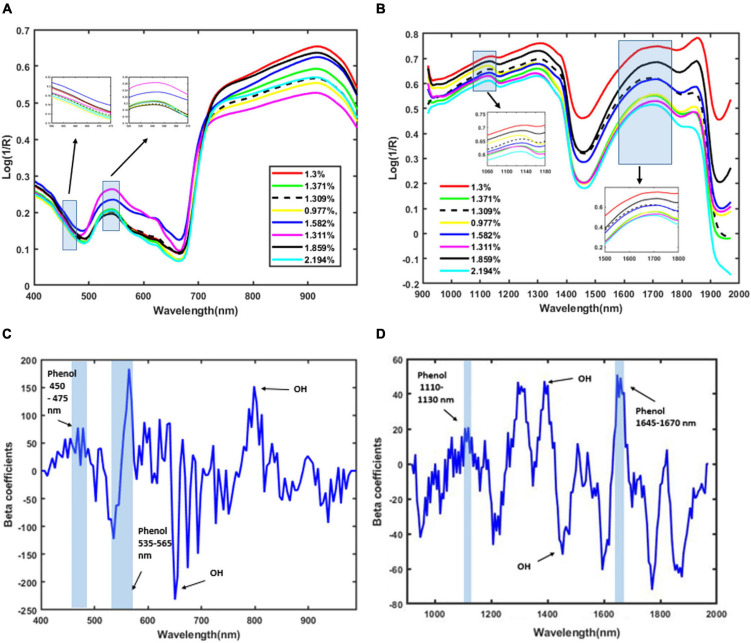
Mean of pre-processed spectra using the second Savitzky–Golay derivative of plants based on total phenolic content. **(A)** VIS/NIR spectra, **(B)** SWIR spectra, **(C)** beta coefficients for VIS/NIR spectra, and **(D)** beta coefficients for SWIR spectra.

Figure 3 also shows a distinct intensity due to the presence of various concentrations of total phenolic compounds in plants under different stress conditions. The OH and CH bonds indicate the main characteristics of phenolic compounds (Frizon et al., 2015). In the VIS/NIR spectra, there is a peak in the beta coefficients in the wavelength from 450 to 475 nm and from 535 to 565 nm. These regions are related to the phenolic compounds (Mayranti et al., 2019). In the SWIR spectra, there is a second CH overtone band that arises in the band range between 1,110 and 1,130 nm and a first CH overtone band that arises in the band range between 1,645 and 1,670 nm. Our model also predicts the presence of OH in the wavelength region between 650 and 780 nm for the VIS/NIR spectra, matching the findings in Zhang et al. (2012) and Jopia et al. (2020). Similarly, the OH prediction at the 1,205 and 1,450 nm bands of the SWIR images matches the findings from Frizon et al. (2015) and Yan et al. (2021).

### Model performance

There are various studies related to the phenolic compounds and plant stress. Sytar et al. (2020) analyzed the hyperspectral and multispectral fluorescence spectra related to the phenolic compounds in chicory leaves. Liu et al. (2019) studied about the total phenolics in *Flos Lonicerae*, grown without any stress, using HSI. *Flos Lonicerae* is a Chinese medicinal herb and their quality is assessed by the total phenolic compounds which acts as one of the quality factors. In their study, they achieved the prediction accuracy of above 96% with different preprocessing methods. The antioxidant activity, polyphenols and fermentation index were identified in single cocoa beans using HSI (Caporaso et al., 2018b). They had obtained the total phenolic compound prediction accuracy of 70%. In addition, Sytar et al. (2019) studied the phenolic compounds accumulation in plants exposed to different colors. They analyzed the correlation indices between the spectral reflectance parameters and the phenolic compounds. Similarly, there are various studies focus on analyzing the chemical compounds in plants and food products (Wold et al., 2016; Morales-Sillero et al., 2018; Shrestha et al., 2020; Amanah et al., 2021). These studies provide an opportunity to analyze the SWIR and VIS/NIR spectra and measure and analyze the changes in phenolic compounds in plants grown under various stress conditions at the same time. In our study, the SWIR and VIS/NIR spectra are analyzed for their changes in various stress conditions, and also the relationship of spectral changes and stress conditions to the changes in total phenolic contents are analyzed.

**FIGURE 4:**
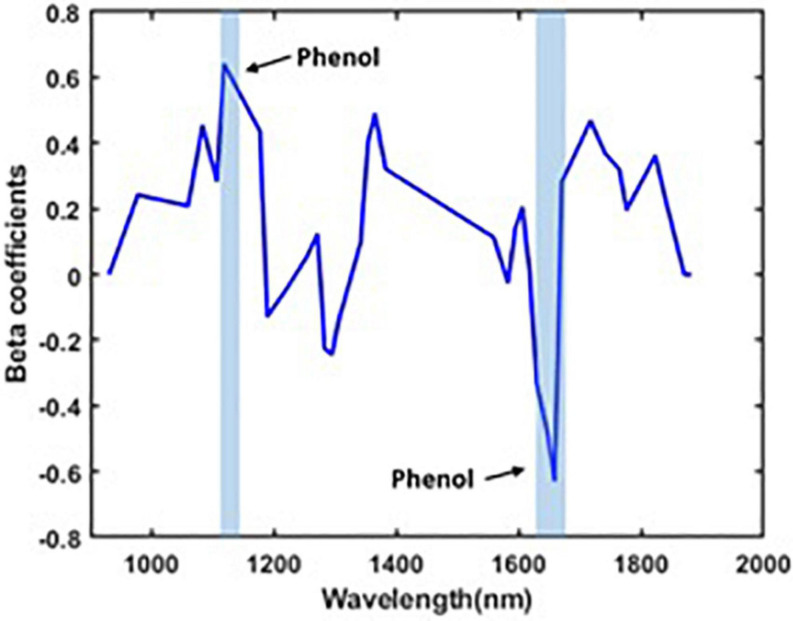
Beta coefficients for SWIR spectra after waveband reduction.

The performance of the developed model is discussed as follows. The number of wavebands that has been selected after waveband reduction is 35 including the wavebands corresponding to the wavelength that represents the phenolic compounds (1,112, 1,118, 1,135, 1,646, 1,652, 1,658, 1,664, and 1,670 nm). The beta coefficients of the PLSR model after waveband reduction is given in Figure 4. The performance of the PLSR model (over reduced waveband spectra) was also compared to other regression models, such as the support vector machine (SVM), regression tree (RegTree), and principal component regression (PCR) models, and is shown in Table 4.

**TABLE 4:** Comparison of PLSR model performance with other regression models.

Method	R_*c*_^2^	SEC	R_*v*_^2^	SEP
SVM	0.94	0.10	0.89	0.14
RegTree	0.92	0.12	0.86	0.16
PCR	0.93	0.11	0.91	0.12
PLSR	0.97	0.05	0.96	0.07

**FIGURE 5:**
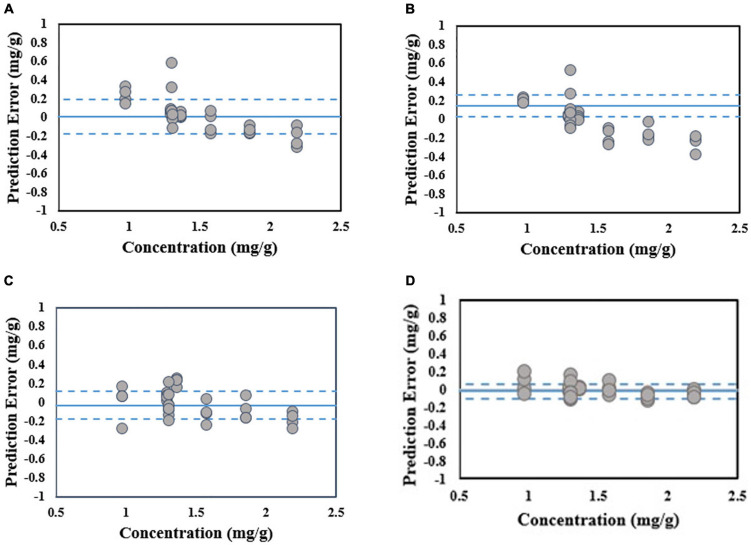
Scatter plot of phenolic concentration reference value versus prediction errors for validation dataset. The average value and the lines of average ± one standard deviation of prediction error are shown in solid and dashed blue lines respectively. **(A)** SVM, **(B)** RegTree, **(C)** PCR, and **(D)** PLSR.

It can be observed from Table 4 that the standard error of prediction (SEP) value of the PLSR model is closer to zero (0.07 mg/g) when compared to the other models. Additionally, the *R*^2^-value of the PLSR model is higher than the other models. These show that the total phenolic compounds for all of the stress conditions are predicted accurately by the PLSR model. On contrary, the other regression methods that are mentioned in Table 4 produce *R*^2^-values that are comparatively lower and higher SEP values. This shows that the performance of those methods is not uniform over the various stress conditions. In that sense, the PLSR model is capable of predicting the total phenolic compounds better than the other models mentioned in Table 4. It can be observed from Figure 5 that the PLSR model is less biased toward the under-prediction or over-prediction of total phenolic compounds, and they are predicted accurately among these models despite various kinds of stress. Overall, the experiment results show that the PLSR model is better able to predict the total phenolic compounds when compared to the other models given in Table 4.

### Visualization image based on phenolic content in Arabidopsis

The advantage of HSI in chemometrics is its ability to generate the chemical compound distribution in the samples. Many researchers have already proven that compound visualization is possible with HSI. Amanah et al. (2021) successfully used HSI and visualized the content of anthocyanins in black rice powder and seeds. The protein in peanuts (Yu et al., 2017) and the fat and moisture content in Atlantic solmon (Zhu et al., 2014) were visualized using HSI and chemometrics. In addition, the hyperspectral images were used to produce the chemical images for visualizing the chemical distribution in peanut samples using the image analysis algorithm and pseudo color operation (Cheng et al., 2018). Wen et al. (2019) visualized the distribution of chlorophyll contents in apple leaves. In general, the advantage of the chemical images is to recognize the chemical changes in the samples by the changed color distribution. In our study, the chemical images are helpful in visualizing the phenolic compounds of Arabidopsis plants grown under different stress conditions.

**FIGURE 6:**
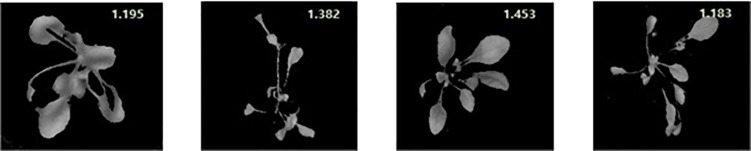
Total phenolic content prediction of individual plants in a growing condition (RedBlue+Drought). The predicted value is given in the top-right corner of each image.

**FIGURE 7:**
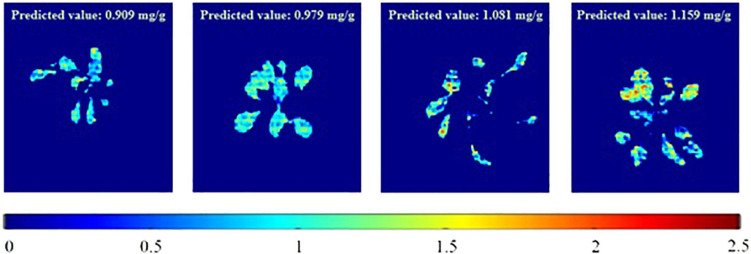
Total phenolic content prediction of individual plants in a certain growing (stress) condition (RedBlue+Non-Drought).

**FIGURE 8:**
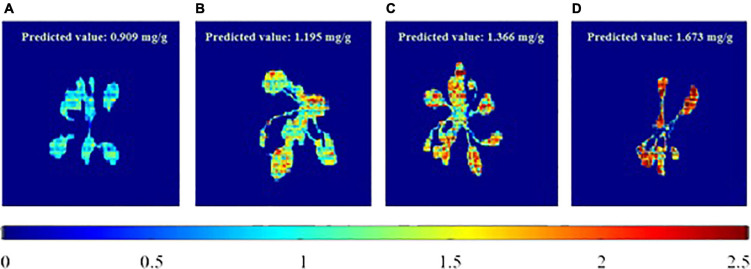
Total phenolic content prediction of plants in various growing (stress) conditions. **(A)** RedBlue+Non-Drought, **(B)** RedBlue+Drought, **(C)** Blue+Non-Drought and **(D)** Red+Drought.

**FIGURE 9:**
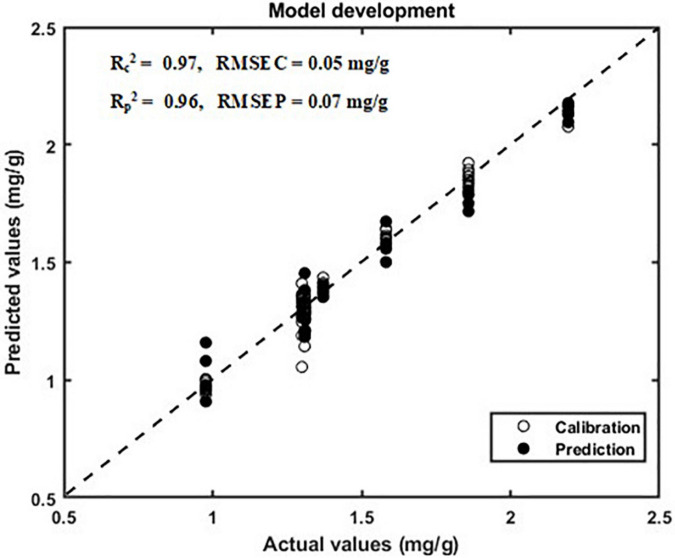
Prediction plot of total phenolic compounds in plants under various stress conditions.

To visualize the total phenolic content in the plant, the chemical images that were obtained from the PLSR model have been generated. These images provide the spatial distribution and concentrations, which help to determine the presence of phenolic compounds. The total concentrations of the phenolic compounds that are present in the sample are clearly confirmed, and the color change from blue to red indicates the increase in the total concentrations of the phenolic compounds. Figure 6 shows the total phenolic content prediction. Figure 7 shows the chemical images of the plants grown in one condition. The colors of the plants grown within this condition show some slight variations, indicating the variations in the total phenolic contents among them. The phenolic compounds are not uniformly distributed in the, affecting the sample homogeneity. In this study, the total phenolic content was examined based on the various growing (either light or drought stress or combination of both) conditions. The evaluation of the total phenolic compounds could not be conducted for each plant because of the minimum weight requirement for HPLC analysis. Hence, to justify the prediction on a single plant, the mean concentration of the total phenolic compounds for each growing condition was calculated, and Figure 7 shows the prediction for plants grown in a condition. The mean predicted value was compared to the reference value that was obtained from HPLC. To evaluate the prediction accuracy, the root mean square (RMSE) was calculated and was shown to be equal to 0.11 mg/g (similar to standard error). Figures 7, 8 show the chemical images of the plants grown in one condition and under various stress conditions, respectively. It can be observed from Figure 8 that the color of the plants shows drastic variations that represent the variations in the total phenolic content of the plants. It is evident that the stress effects can be identified through these drastic variations in the chemical compounds. A prediction plot for all of the growing conditions is given in Figure 9.

### Effects of stresses on total phenolic contents in Arabidopsis

Many studies were conducted to identify the phenolic content and other chemical changes in plants due to stresses. Posmyk et al. (2005) observed the changes in antioxidant enzymes and isoflavonoids in chilled soybean seedlings. The changes in phenolic compounds were studied in response to Phytophthora ramorum infection (Ockels et al., 2007). The changes in phenolic compounds and antioxidant properties were observed in grapevine under drought stress and followed by recovery (Weidner et al., 2009). Similarly, Król et al. (2014) analyzed the phenolic compounds changes and antioxidant properties in grapevine grown under long-term drought stress.

**FIGURE 10:**
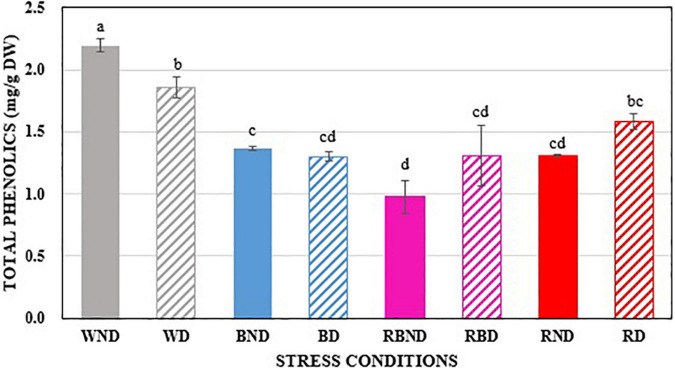
The comparison of total phenolic compounds in plants under various stress environments. The different letters above the error bars denote the significant difference (*p* < 0.005) between the parameter areas using Duncan’s multiple range test (*n = 3*, mean ± SD).

In our study, we observed the total phenolic compounds response under various stress conditions. Arabidopsis plants were grown under stresses such as various LED lights, drought, and in combinations of the different LED light sources and drought, as explained in the Materials and Methods section. Then, their total phenolic contents were predicted using HSI image analysis. The concentrations of the total phenolic contents were affected by the different stress environments, the effects of which are shown in Figure 10. It can be observed from Figure 10 that the total phenolic contents have reduced been by the stress environments (WD: white + drought; BND: blue + non-drought; BD: blue + drought; RBND: red–blue + non-drought; RBD: red–blue + drought; RND: red + non-drought; RD: red + drought) when compared to the plants that had been grown in the control environment (WND: white + non-drought). It can also be observed that the total phenolic content is high in drought conditions in the presence of red light (RBD and RD) when compared to the corresponding non-drought conditions (RBND and RD). However, the total phenolic content is lower in drought conditions in the absence of red light (WND and BND) when compared to the corresponding non-drought conditions (WND and BND). In terms of the morphological structure of the plants, the canopy was increased by blue light, and the height was increased by the red light when compared to the control conditions. Additionally, the red–blue light was shown to have the morphological effects (height and canopy) of both the red and blue lights. This shows that the red–blue light and red light can be used as a pre-treatment for plants that will be grown in drought conditions, which matches with the results that were determined in the research work (Ahmadi et al., 2020). Selecting the appropriate wavelengths for LED light that facilitate the plant growth requires further research.

## Conclusion

In our study, a non-destructive and rapid method for predicting the total phenolic compounds in Arabidopsis plants using VIS/NIR and SWIR image analysis is reported. In addition, various LED and drought stresses were imparted upon the plants, and the phenolic prediction was examined. HSI combined with the PLSR model shows high prediction performance, with *R*^2^-values of 0.94 and 0.93 for the calibration and validation, respectively, for the spectra that were extracted from the segmented images from the SWIR region, and *R*^2^-values of 0.87 and 0.83 for the calibration and validation, respectively, for the spectra that were extracted from the segmented images from the VIS/NIR region. The second Savitzky–Golay (SG) derivative pre-processing method resulted in high prediction performance, with and *R*^2^-value of 0.93 and the lowest SEP value of 0.11 mg/g when compared to raw and other preprocessing methods. Since the SWIR spectra yields better prediction results, it is used for further analysis. After waveband reduction, the PLSR model attains the *R*^2^-values of 0.97 and 0.96 for the calibration and validation, respectively. This performance was also compared with different state-of-the-art machine learning methods. The prediction performance of the PLSR with the SG-2nd derivative after waveband reduction showed better performance than all of those models. The chemical images confirm the presence of phenolic compounds in the Arabidopsis plants. This study shows the potential of HSI to predict and helps in analysis of the total phenolic compounds in plants under various stress conditions. The HSI approach has the potential of rapid analysis and demonstrates the possibility of developing an automatic total phenolic prediction mechanism.

## Data availability statement

The original contributions presented in this study are included in the article/supplementary material, further inquiries can be directed to the corresponding author.

## Author contributions

PJ and B-KC contributed to conception and design of the study. RS, BV, and SP organized the database. PJ, RJ, and MF performed the statistical analysis. PJ wrote the first draft of the manuscript. DS and B-KC wrote sections of the manuscript. All authors contributed to manuscript revision, read, and approved the submitted version.

## References

[B1] Ahmadi T. Shabani L. Sabzalian M. R. (2020). LED light mediates phenolic accumulation and enhances antioxidant activity in *Melissa officinalis* L. under drought stress condition. *Protoplasma* 257 1231–1242. 32342193 10.1007/s00709-020-01501-4

[B2] Amanah H. Z. Wakholi C. Perez M. Faqeerzada M. A. Tunny S. S. Masithoh R. E. (2021). Near-infrared hyperspectral imaging (NIR-HSI) for nondestructive prediction of anthocyanins content in black rice seeds. *Appl. Sci.* 11:4841. 10.3390/app11114841

[B3] Aenugu H. P. R. Kumar D. S. Srisudharson N. P. Ghosh S. S. Banji D. (2011). Near infra red spectroscopy—An overview. *Int. J. Chemtech Res.* 3 825–836.

[B4] Asaari M. S. M. Mertens S. Dhondt S. Inzé D. Wuyts N. Scheunders P. (2019). Analysis of hyperspectral images for detection of drought stress and recovery in maize plants in a high-throughput phenotyping platform. *Comput. Electron. Agric.* 162 749–758. 10.1016/j.compag.2019.05.018

[B5] Boughton B. A. Thinagaran D. Sarabia D. Bacic A. Roessner U. (2016). Mass spectrometry imaging for plant biology: a review. *Phytochem Rev.* 15 445–488. 10.1007/s11101-015-9440-2 27340381 PMC4870303

[B6] Candolfi A. De Maesschalck R. Jouan-Rimbaud D. Hailey P. A. Massart D. L. (1999). The influence of data pre-processing in the pattern recognition of excipients near-infrared spectra. *J. Pharm. Biomed.* 21 115–132. 10.1016/S0731-7085(99)00125-9 10701919

[B7] Caporaso N. Whitworth M. B. Fisk I. D. (2018a). Protein content prediction in single wheat kernels using hyperspectral imaging. *Food Chem.* 240 32–42. 10.1016/j.foodchem.2017.07.048 28946278 PMC5625851

[B8] Caporaso N. Whitworth M. B. Fowler M. S. Fisk I. D. (2018b). Hyperspectral imaging for non-destructive prediction of fermentation index, polyphenol content and antioxidant activity in single cocoa beans. *Food Chem.* 258 343–351. 10.1016/j.foodchem.2018.03.039 29655743 PMC5914545

[B9] Cheng J. H. Jin H. Liu Z. (2018). Developing a NIR multispectral imaging for prediction and visualization of peanut protein content using variable selection algorithms. *Infrared Phys. Technol.* 88 92–96. 10.1016/j.infrared.2017.11.018

[B10] Choi J. Y. Heo S. Bae S. Kim J. Moon K. D. (2020). Discriminating the origin of basil seeds (*Ocimum basilicum* L.) using hyperspectral imaging analysis. *LWT* 118:108715. 10.1016/j.lwt.2019.108715

[B11] Dao P. D. He Y. Proctor C. (2021). Plant drought impact detection using ultra-high spatial resolution hyperspectral images and machine learning. *Int. J. Appl. Earth Obs. Geoinf.* 102:102364. 10.1016/j.jag.2021.102364

[B12] Del Rio D. Rodriguez-Mateos A. Spencer J. P. Tognolini M. Borges G. Crozier A. (2013). Dietary (poly) phenolics in human health: structures, bioavailability, and evidence of protective effects against chronic diseases. *Antioxid. Redox Signal.* 18 1818–1892. 10.1089/ars.2012.4581 22794138 PMC3619154

[B13] Erkinbaev C. Derksen K. Paliwal J. (2019). Single kernel wheat hardness estimation using near infrared hyperspectral imaging. *Infrared Phys. Technol.* 98 250–255. 10.1016/j.infrared.2019.03.033

[B14] Frizon C. N. Oliveira G. A. Perussello C. A. Peralta-Zamora P. G. Camlofski A. M. Rossa ÜB. (2015). Determination of total phenolic compounds in yerba mate (*Ilex paraguariensis*) combining near infrared spectroscopy (NIR) and multivariate analysis. *LWT* 60 795–801. 10.1016/j.lwt.2014.10.030

[B15] Gong Z. G. Hu J. Wu X. Xu Y. J. (2017). The recent developments in sample preparation for mass spectrometry-based metabolomics. *Crit. Rev. Anal. Chem.* 47 325–331. 10.1080/10408347.2017.1289836 28631936

[B16] Han Z. Gao J. (2019). Pixel-level aflatoxin detecting based on deep learning and hyperspectral imaging. *Comput. Electron. Agric.* 164:104888. 10.1016/j.compag.2019.104888

[B17] Ignat I. Volf I. Popa V. I. (2011). A critical review of methods for characterisation of polyphenolic compounds in fruits and vegetables. *Food Chem.* 126 1821–1835. 10.1016/j.foodchem.2010.12.026 25213963

[B18] Jopia A. Zambrano F. Pérez-Martínez W. Vidal-Páez P. Molina J. De la Hoz Mardones F. (2020). Time-series of vegetation indices (VNIR/SWIR) derived from Sentinel-2 (A/B) to assess turgor pressure in kiwifruit. *ISPRS. Int. J. Geo-Inf.* 9:641. 10.3390/ijgi9110641

[B19] Kokaly R. F. Skidmore A. K. (2015). Plant phenolics and absorption features in vegetation reflectance spectra near 1.66 μm. *Int. J. Appl. Earth Obs. Geoinf.* 43 55–83. 10.1016/j.jag.2015.01.010

[B20] Kresta J. V. Marlin T. E. MacGregor J. F. (1994). Development of inferential process models using PLS. *Comput. Chem. Eng.* 18 597–611. 10.1016/0098-1354(93)E0006-U

[B21] Król A. Amarowicz R. Weidner S. (2014). Changes in the composition of phenolic compounds and antioxidant properties of grapevine roots and leaves (*Vitis vinifera* L.) under continuous of long-term drought stress. *Acta Physiol. Plant* 36 1491–1499. 10.1007/s11738-014-1526-8

[B22] Liang K. Liu Q. X. Xu J. H. Wang Y. Q. Okinda C. S. Shena M. X. (2018). Determination and visualization of different levels of deoxynivalenol in bulk wheat kernels by hyperspectral imaging. *J. Appl. Spectrosc.* 85 953–961. 10.1007/s10812-018-0745-y

[B23] Liu C. Huang W. Yang G. Wang Q. Li J. Chen L. (2020). Determination of starch content in single kernel using near-infrared hyperspectral images from two sides of corn seeds. *Infrared Phys. Technol.* 110:103462. 10.1016/j.infrared.2020.103462

[B24] Liu R. H. (2013). Health-promoting components of fruits and vegetables in the diet. *Adv. Nutr.* 4 384S–392S. 10.3945/an.112.003517 23674808 PMC3650511

[B25] Liu Y. Wang Q. Gao X. Xie A. (2019). Total phenolic content prediction in Flos Lonicerae using hyperspectral imaging combined with wavelengths selection methods. *J. Food Proc. Eng.* 42:13224. 10.1111/jfpe.13224

[B26] Ma L. Peng Y. Pei Y. Zeng J. Shen H. Cao J. (2019). Systematic discovery about NIR spectral assignment from chemical structural property to natural chemical compounds. *Sci. Rep.* 9 1–17. 10.1038/s41598-019-45945-y 31263130 PMC6603013

[B27] Mayranti F. P. Saputro A. H. Handayani W. (2019). “Wavelength Selection of Persimmon Leafusing Decision Tree Method in Visible Near-Infrared Imaging,” in *proceedings of the 2019 International Conference on Advanced Computer Science and information Systems (ICACSIS)*, Bali: IEEE, 113–118.

[B28] Mertens S. Verbraeken L. Sprenger H. Demuynck K. Maleux K. Cannoot B. (2021). Proximal hyperspectral imaging detects diurnal and drought-induced changes in maize physiology. *Front. Plant Sci.* 12:640914. 10.3389/fpls.2021.640914 33692820 PMC7937976

[B29] Morales-Sillero A. Pierna J. A. F. Sinnaeve G. Dardenne P. Baeten V. (2018). Quantification of protein in wheat using near infrared hyperspectral imaging: Performance comparison with conventional near infrared spectroscopy. *J. Near Infrared Spectrosc.* 26 186–195. 10.1364/JNIRS.26.000186

[B30] Ockels F. S. Eyles A. McPherson B. A. Wood D. L. Bonello P. (2007). Phenolic chemistry of coast live oak response to *Phytophthora ramorum* infection. *J. Chem. Ecol.* 33 1721–1732. 10.1007/s10886-007-9332-z 17713820

[B31] Osborne B. G. Fearn T. Hindle P. H. (1993). *Practical NIR spectroscopy with applications in food and beverage analysis.* London: Longman scientific and technical.

[B32] Park C. H. Yeo H. J. Park S. Y. Kim J. K. Park S. U. (2019). Comparative phytochemical analyses and metabolic profiling of different phenotypes of Chinese cabbage (*Brassica rapa* ssp. *pekinensis*). *Foods* 8:587. 10.3390/foods8110587 31752320 PMC6915346

[B33] Pires N. M. M. Dong T. Hanke U. Hoivik N. (2014). Recent developments in optical detection technologies in lab-on-a-chip devices for biosensing applications. *Sensors* 14 15458–15479. 10.3390/s140815458 25196161 PMC4178989

[B34] Posmyk M. M. Bailly C. Szafrañska K. Janas K. M. Corbineau F. (2005). Antioxidant enzymes and isoflavonoids in chilled soybean (*Glycine max* (L.) Merr.) seedlings. *J. Plant Physiol.* 162 403–412. 10.1016/j.jplph.2004.08.004 15900882

[B35] PraveenKumar J. Domnic S. (2020). Rosette plant segmentation with leaf count using orthogonal transform and deep convolutional neural network. *Mach. Vis. Appl.* 31 1–14. 10.1007/s00138-019-01056-2

[B36] Seca A. M. Pinto D. C. (2018). Plant secondary metabolites as anticancer agents: successes in clinical trials and therapeutic application. *Int. J. Mol. Sci.* 19:263. 10.3390/ijms19010263 29337925 PMC5796209

[B37] Shrestha L. Kulig B. Moscetti R. Massantini R. Pawelzik E. Hensel O. (2020). Comparison between hyperspectral imaging and chemical analysis of polyphenol oxidase activity on fresh-cut apple slices. *J. Spectrosc.* 2020 1–10. 10.1155/2020/7012525 PMC7022590 31936660

[B38] Şirin S. Aslım B. (2019). Determination of antioxidant capacity, phenolic acid composition and antiproliferative effect associated with phenylalanine ammonia lyase (PAL) activity in some plants naturally growing under salt stress. *Med. Chem. Res.* 28 229–238. 10.1007/s00044-018-2278-6

[B39] Sturtevant D. Lee Y. J. Chapman K. D. (2016). Matrix assisted laser desorption/ionization-mass spectrometry imaging (MALDI-MSI) for direct visualization of plant metabolites in situ. *Curr. Opin. Biotechnol.* 37 53–60. 10.1016/j.copbio.2015.10.004 26613199

[B40] Sytar O. Zivcak M. Neugart S. Brestic M. (2020). Assessment of hyperspectral indicators related to the content of phenolic compounds and multispectral fluorescence records in chicory leaves exposed to various light environments. *Plant Physiol. Biochem.* 154 429–438. 10.1016/j.plaphy.2020.06.027 32912483

[B41] Sytar O. Zivcak M. Neugart S. Toutounchi P. M. Brestic M. (2019). Precultivation of young seedlings under different color shades modifies the accumulation of phenolic compounds in Cichorium leaves in later growth phases. *Environ. Exp. Bot.* 165 30–38. 10.1016/j.envexpbot.2019.05.018

[B42] Tan W. Sun L. Yang F. Che W. Ye D. Zhang D. (2018). Study on bruising degree classification of apples using hyperspectral imaging and GS-SVM. *Optik* 154 581–592. 10.1016/j.ijleo.2017.10.090

[B43] Tian X. Y. Aheto J. H. Bai J. W. Dai C. Ren Y. Chang X. (2021). Quantitative analysis and visualization of moisture and anthocyanins content in purple sweet potato by Vis–NIR hyperspectral imaging. *J. Food Proc. Preserv.* 45:5128. 10.1111/jfpp.15128

[B44] Tsao R. (2010). Chemistry and biochemistry of dietary polyphenols. *Nutrients* 2 1231–1246. 10.3390/nu2121231 22254006 PMC3257627

[B45] Wang Q. Liu Y. Gao X. Xie A. Yu H. (2019). Potential of hyperspectral imaging for nondestructive determination of chlorogenic acid content in Flos Lonicerae. *J. Food Meas. Charact.* 13 2603–2612. 10.1007/s11694-019-00180-x

[B46] Weidner S. Karolak M. Karamac M. Kosinska A. Amarowicz R. (2009). Phenolic compounds and properties of antioxidants in grapevine roots [*Vitis vinifera* L.] under drought stress followed by recovery. *Acta Soc. Bot. Pol.* 78 97–103. 10.5586/asbp.2009.036

[B47] Wen X. Zhu X. Yu R. Xiong J. Gao D. Jiang Y. (2019). Visualization of chlorophyll content distribution in apple leaves based on hyperspectral imaging technology. *Agric. Sci.* 10 783–795. 10.4236/as.2019.106060

[B48] Wold J. P. Kermit M. Segtnan V. H. (2016). Chemical imaging of heterogeneous muscle foods using near-infrared hyperspectral imaging in transmission mode. *Appl. Spectrosc.* 70 953–961. 10.1177/0003702816641260 27257302

[B49] Yan Y. Ren J. Tschannerl J. Zhao H. Harrison B. Jack F. (2021). Nondestructive phenolic compounds measurement and origin discrimination of peated barley malt using near-infrared hyperspectral imagery and machine learning. *IEEE. Trans. Instrum. Meas.* 70 1–15. 10.1109/TIM.2021.308227433776080

[B50] Yu H. W. Wang Q. Shi A. M. Yang Y. Liu L. Hu H. (2017). Visualization of protein in peanut using hyperspectral image with chemometrics. *Spectrosc. Spect. Anal.* 37 853–858. 10.3964/j.issn.1000-0593201703-0853-06 30160400

[B51] Zhang N. Liu X. Jin X. Li C. Wu X. Yang S. (2017). Determination of total iron-reactive phenolics, anthocyanins and tannins in wine grapes of skins and seeds based on near-infrared hyperspectral imaging. *Food Chem.* 237 811–817. 10.1016/j.foodchem.2017.06.007 28764071

[B52] Zhang Q. Li Q. Zhang G. (2012). Rapid determination of leaf water content using VIS/NIR spectroscopy analysis with wavelength selection. *Spectrosc-Int. J.* 27 93–105. 10.1155/2012/276795

[B53] Zhu F. Zhang H. Shao Y. He Y. Ngadi M. (2014). Mapping of fat and moisture distribution in Atlantic salmon using near-infrared hyperspectral imaging. *Food Bioproc. Tech.* 7 1208–1214. 10.1007/s11947-013-1228-z

